# Perceived stressors and coping mechanisms of female migrant domestic workers in Singapore

**DOI:** 10.1371/journal.pone.0210717

**Published:** 2019-03-20

**Authors:** Tine Van Bortel, Steven Martin, Sabrina Anjara, Laura B. Nellums

**Affiliations:** 1 Institute for Health and Human Development, University of East London, London, United Kingdom; 2 Cambridge Institute of Public Health, School of Clinical Medicine, University of Cambridge, Cambridge, United Kingdom; 3 Institute for Infection and Immunity, St George’s, University of London, London, United Kingdom; 4 Section of Infectious Diseases and Immunity, Department of Medicine, Imperial College London, London, United Kingdom; Temple University, UNITED STATES

## Abstract

**Introduction:**

Worldwide, there are between 50–67 million migrant domestic workers, the majority of whom are women. In many countries, provisions are not in place to protect female migrant domestic workers. These women may be at risk of occupational and social stressors, including exploitation and abuse, which may negatively impact on their quality of life, including psychological health. Research examining the occupational, social, and psychological needs of FMDWs from a public health perspective is critical to guide the development of policies which ensure wellbeing, prevent abuse, and align with international priorities to improve population health. Though there have been a number of high-profile incidents of exploitation and abuse, there has been limited research on the stressors experienced by these communities, their perceived impact, or coping mechanisms.

**Materials and methods:**

Thematic analysis was used to analyse qualitative free-text written responses collected as part of a cross-sectional survey on the relationship between social and occupational stressors and the health and quality of life of FMDWs in Singapore. Responses correspond to open-ended questions in the qualitative component of the survey examining three domains: causes of stress, coping strategies, and what people can do to help with stress.

**Results:**

Responses from 182 FMDWs were analysed. Key themes were identified around causes of stress (including ‘work and agency’, ‘the pervasiveness of financial need’, and ‘family and obligation’), coping strategies, and social support. Each theme describes key factors which contribute to the occupational and social stressors experienced and reported by FMDWs.

**Discussion:**

This research highlights the stressors FMDWs in Singapore experience, as well as key coping mechanisms. There is a clear need for policies which facilitate FMDWs’ ability to utilise these coping resources, and which protect against coercive or exploitative employment conditions. Strategies are also needed to monitor and evaluate policies intended to protect FMDWs, and to strengthen the implementation of global frameworks targeted at improving workplace conditions and workers’ rights.

## Introduction

There are between 50–67 million migrant domestic workers (MDWs) worldwide [1–5], the majority (between 73.4–83%) of whom are women [2, 4–6]. Migrant domestic workers are employed in low, middle, and high-income countries worldwide, and both south-to-south and south-to-north migration patterns are seen, as there is a demand for labour in both low and high development settings, which increases as individuals migrate, creating a demand for workers in countries of origin.

24% of female migrant domestic workers (FMDWs) are in South-Eastern Asia and the Pacific [2, 4]. Growing numbers of FMDWs globally have contributed to the feminisation of migration [7] and a ‘global care chain’ [8, 9], whereby the demand for labour in more developed countries has been met with the supply of FMDWs from less developed countries [8]. The effects of these processes include creating increased development and deficits in the labour force and carers (e.g. for left behind families) in sending countries [10].

In many countries, provisions are not in place to protect the health or safety of FMDWs, leaving them vulnerable to abuse and exploitation [1]. According to the International Labor Organization [6], 29.9% of MDWs are completely excluded from the scope of national labour laws, 56.6% are not covered by national limitations of normal weekly hours, 44.9% have no entitlement to weekly rest, and 44.4% are excluded from annual leave provisions. Without clear terms of reference, unregulated payment scales and unregistered status, FMDWs are among the most vulnerable groups of workers in the world [11].

Such work environments may increase the risk of exposure to psychosocial hazards including occupational stressors that have been shown to impact on mental and physical health [2, 12, 13–20]. These risks include exposure to physical, sexual, emotional, or financial abuse, financial stressors, exploitation or overwork (including long hours, physically taxing work, lack of days off, or work without fair compensation), and legal stressors associated with immigration status and restrictions relating to their work permits and resident status. Evidence suggests that workers exposed to psychosocial risks, and specifically occupational stressors, may sleep badly, drink excessively, or feel depressed, anxious, nervous or angry due to treatment and working conditions [21, 22].

Previous research has also pointed to the multidimensional stressors that migrant domestic workers, and women in particular, may experience in both the countries in which they are working, and their countries of origin [17,18]. This can include the gendered challenges associated with geographic distance and the division between work and home life that may exist for women [23]. As a result, women who are both migrant domestic workers and have families in their countries of origin may experience stressors linked with isolation and distance from their social networks and cultures [20], (‘mothering from a distance’) [23], and the impact that may be felt by the families that remain in countries of origin (‘left-behind families’) [22].

Despite many resources developed by WHO around health workplaces [24], the impact of workplace factors on the mental and physical health of migrant domestic workers (MDWs) has received inadequate attention [19, 20].

Singapore has a large population of MDWs, with over 230,000 registered work permits [25]. Recent evidence suggests FMDWs in Singapore experience high levels of social isolation and stress, which negatively impact on quality of life. For example, in our previous publication on female migrant domestic workers in Singapore reporting on quantitative data from the cross-sectional survey, we found that nearly 20% of women reported being isolated or very isolated. Isolation was found to be associated with stress, which contributed to worse quality of life [20]. A survey of MDWs in Singapore also identified that 24% had poor mental health, 51% reported verbal abuse, 40% were given less than one rest day per week, and 74% reported restriction of movement [26]. In Singapore, MDWs are excluded from the Employment Act [27], there is no specific legislation for domestic workers nor any compulsory standard unified contracts [4], social protection coverage is on a voluntary basis [3], and foreign domestic workers must also undergo a medical examination and pregnancy test every six months (if a woman is found to be pregnant this can result in immediate repatriation) [28]. These employment conditions ultimately mean these communities are excluded from policies which may prevent their abuse or exploitation [29, 30], which is disproportionately experienced by female workers, or protect their health and quality of life (QoL). Despite prioritisation in global frameworks like the WHO’s Healthy Workplaces global model for action [31–33], there remain significant barriers restricting FMDWs’ rights in Singapore.

Research examining the occupational, social, and psychological needs of FMDWs from a public health perspective is critical to guide the development of policies which ensure wellbeing, prevent abuse, and align with international priorities to improve population health. However, there is insufficient research exploring the needs of FMDWs, their psychological health or access to coping resources [12, 20]. To address these gaps in evidence, this study examines stressors and coping mechanisms experienced by FMDWs in Singapore.

## Materials and methods

This study uses qualitative data from a cross-sectional survey on stressors, social connectivity, coping mechanisms, and QoL among FMDWs in Singapore [20]. The survey was conducted in April 2012, and included FMDWs who: 1) held a Work Permit; 2) were classified as foreign (migrant); and 3) had access to a weekly rest day. Women were invited to participate in the study during their day off, and recruited using convenience and snowball sampling in public places of gathering (e.g. parks). Recruitment approaches and location were determined in collaboration with the President of Transient Workers Count Too, an organisation promoting equitable treatment for migrant workers in Singapore, who has extensive experience working with these groups. This ensured the approaches and locations used were acceptable and appropriate. Informed consent was obtained from all participants, who received an information sheet and were able to ask questions prior to participation. Women were informed that participation would be anonymous and confidential, and that they may elect not to participate or chose to stop participation at any point.

Prior to data collection, the researcher (SGA) engaged with Transient Workers Count Too (TWC2), a civil society organisation working with female migrant domestic workers in Singapore, as well as St. Andrews Cathedral in Singapore, which played a gatekeeping role, facilitating her engagement with migrant domestic workers.

Data were collected by SGA, a female researcher from Indonesia and Singapore, through a written English language survey. The purpose and aims of the study, as well as what it involves, were explained by the researcher. Paper copies of the survey were distributed to women in public places of gathering. Participants were approached in person by the researcher, and identified through convenience and snowball sampling. If women elected to participate, they were able to fill out the paper survey in their own time on their own, and return it to the researcher or gatekeeping organisations.

The survey included an open-ended free-text response section asking women to qualitatively describe: 1) causes of stress, 2) coping strategies, 3) social support, and 4) whether coping strategies and social support helped them to feel less stressed. The selection of these prompts and their use as a framework for the analysis was guided by the evidence-base around the salience of these factors for migrant domestic workers, a related survey being carried out with this community examining these areas [34], and the specific aims of our wider cross-sectional survey to investigate the relationship between stress, social isolation, and the mental health and well-being of female migrant domestic workers [20].

English language proficiency is a requirement for a work permit in Singapore, and all participants thus had proficiency in English to complete the questionnaire and provide fully informed consent. However, translation resources were also available through the multilingual field researcher and gatekeeping organisations. Additional methodological data on the cross-sectional survey can be found elsewhere [20].

Descriptive analyses for the quantitative data from the surveys were carried out using Stata/SE 15.1. Qualitative data from the free-text responses were imported into Excel and Nvivo for data management and analysis, and differences in the socio-demographic characteristics of respondents and non-respondents to the free-text questions analysed in Stata. Qualitative data were analysed using thematic analysis, in line with Braun and Clarke’s (2006) guidelines. Content analysis was also used to quantify the occurrence of key themes identified through the qualitative analysis. The analysis was guided by the framework of the survey, in which participants were asked about causes of stress, coping strategies and social support. Codes and themes within each of these components of the framework were derived from the data and developed through an iterative process by two researchers (LBN and TVB). During this process, saturation was achieved. Researchers developed the coding framework and themes through a collaborative and iterative process. This discursive co-production approach supported the quality and rigour of the analysis and trustworthiness of the results by comparing codes and themes, and reaching a consensus on the results of the analysis. All authors were immersed in the data and involved in the analysis throughout the project to increase rigour. Throughout the research, a process of active reflexivity (fieldnotes; assessing and documenting the researchers’ perspectives in the research) was undertaken in an effort to examine the authors’ roles and influence on the interpretative process, which is recognised as a key part of improving the quality and rigour of qualitative research. The importance of this process of active reflexivity throughout the analysis is situated within a constructivist philosophy underpinning the research, whereby the knowledge or ‘findings’ identified through the data collection and analysis are informed by the active involvement of not only the participant, but also the researcher in generating data, which is informed by the lived experience of both.

The codes and themes identified from the data were examined across participant demographics, however all themes were found to be described across all groups in relation to country of origin, religion, age, and work experience. Themes within each of the core areas (causes of stress, coping, and social support) are presented in italics. Sub-themes are presented with single quotation marks, and participant quotes are indicated using double quotes.

Data collected in this study can be obtained by contacting the authors, and is also presented in S1 Table.

### Ethics, consent and permissions

Full ethical approval was granted by the King’s College London Psychiatry, Nursing and Midwifery Research Ethics Committee on 3 April 2012 PNM/11/12-77. Written informed consent was obtained from all participants, and confirmed through submission of the written survey. As this was a community-based study and not a health services study, ethical approval was not needed from medical or health bodies in Singapore; no patients or health services were involved and the study only engaged community members.

## Results

In total, 220 surveys were distributed, and 182 women elected to participate (82.7% response rate). Of these, 100 women responded to the free-text questions, and were included in the qualitative analysis.

### Socio-demographic characteristics

The mean age of participants was 35.1 years (SD 7.6, range 20–63). The mean number of months of work experience was 89.9 (SD 72.9, range 1–432). Participants were from the Philippines (n = 104), Indonesia (n = 68), Myanmar (n = 9), and Sri Lanka (n = 1), and identified as Christian (n = 117), Muslim (n = 56), Buddhist (n = 6), Sikh (n = 1), or as ‘freethinkers’ (n = 2) (Table 1). There were no differences in the socio-demographic characteristics of participants who responded to the free-text questions and those who did not, with the exception of months of work experience, which was less among women who responded (mean 74.5, sd 59.1 vs 107.2, sd 84.5 respectively; OR: 1.01, 95% CI: 1.00–1.01; p = 0.005).

**Table 1:** Participant characteristics (n = 182).

Characteristics	N	%
*Socio-demographic characteristics*		
Age (2 missing)		
<30	50	27.8
30–39	83	46.1
40–49	38	21.1
50+	9	5.0
Country of origin		
The Philippines	104	57.1
Indonesia	68	37.4
Myanmar	9	4.9
Sri Lanka	1	0.5
Marital status (1 missing)		
Single	79	43.6
Married	75	41.4
Widowed/Divorced	27	14.9
Education (5 missing)		
Primary/secondary	108	61.0
Diploma/university	69	39.0
Religion		
Catholic	76	41.8
Christian (excl. Catholic)	41	22.5
Buddhist/Sikh	7	3.8
No religion	2	1.1
*Employment characteristics*		
First job		
Yes	96	52.7
No	86	47.3
Work experience (years (5 missing)		
≤2	24	13.6
2–6	71	40.1
6–10	36	20.3
10–20	41	23.2
>20	5	2.8
Previous country of work (n = 68)		
Home country	29	33.7
Arabian Peninsula	20	23.2
Hong Kong/Taiwan	17	19.8
Singapore	10	11.6
Malaysia/Brunei	9	10.5
North America	1	1.2

Key themes identified in the research were identified across these diverse groups of FMDWs, highlighting the shared experience among these women.

### Causes of stress

**Fig 1:**
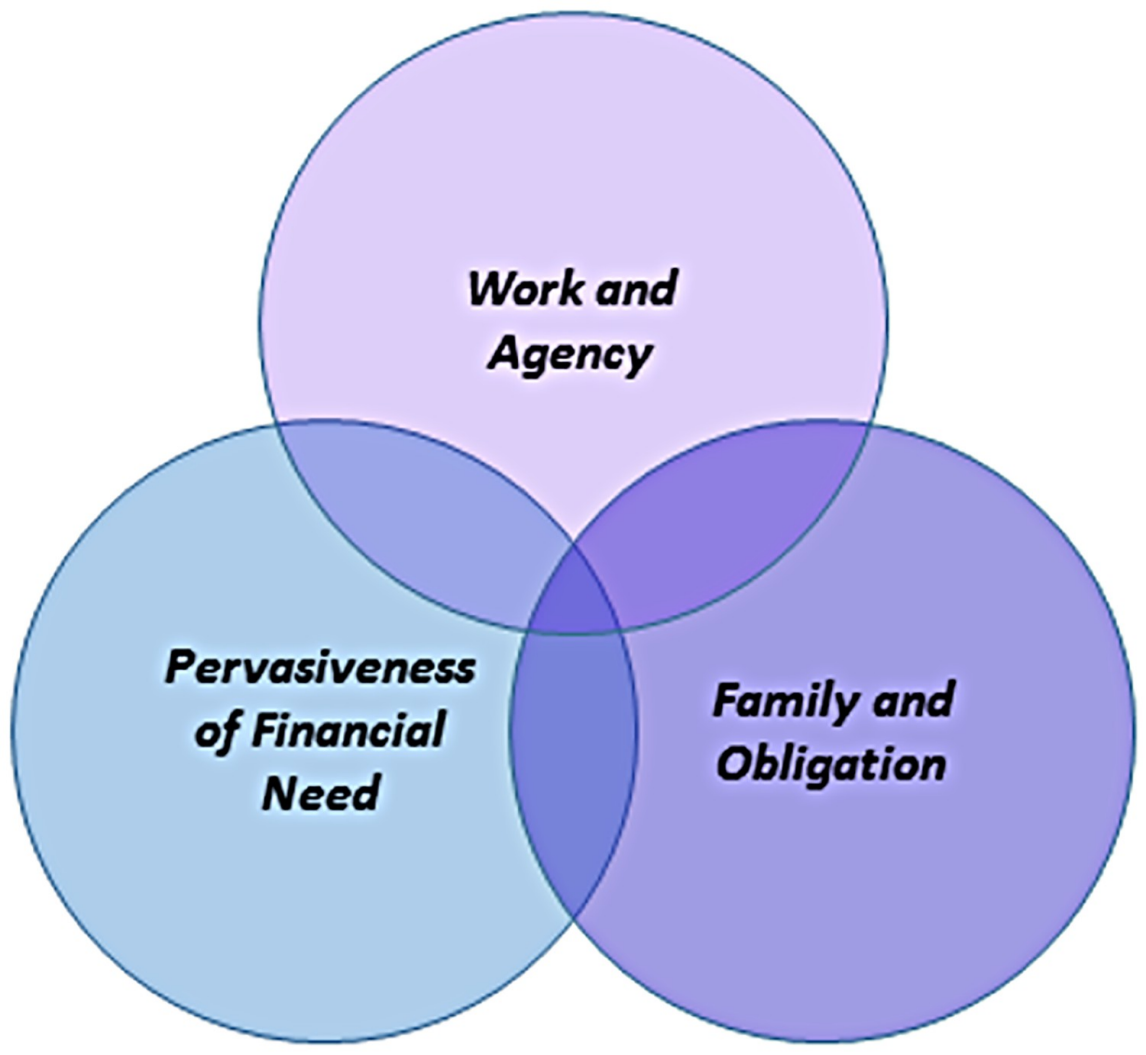
Causes of stress: Sub-themes.

Three interrelated and hierarchical themes were identified describing the overarching causes of stress women experienced: *work and agenc*y; *the pervasiveness of financial need*; and *family and obligation* (Fig 1). *Work and agency* featured at the top of the hierarchy of causes of stress women experienced, encompassing and contributing to the stress then experienced in the other interrelated areas (*the pervasiveness of financial need*, *and family and obligation)*.

#### Work and agency

Participants consistently reported work was a primary source of stress, and the limited agency they felt they often had in their workplaces, exacerbating the stresses associated with work, and contributing to the other stressors (*pervasiveness of financial need* and *family and obligation*). This theme included the sub-themes: ‘power inequalities in relation to their superiors’; ‘being subject to their employment contract and the demands of the job’; and ‘insecurity in relation to their employment’.

Participants described ‘the power inequalities they experienced in relation to their superiors’ and the resulting power hierarchies that existed in their employment: “Sometimes my duty to serve my employer’s family make me feel stressed (participant 103).” In particular, they reported the stresses they experienced because employers were “in a bad mood (participant 115)”, which included “job pressure and unreasonable decision, expectation of high standard (participant 109)”.

Respondents also described the stresses associated with ‘being subject to their employment contract and the demands of the job’, which encompassed the restrictions associated with their work visas as migrant domestic workers, and the corresponding requirements of their employment contract to which their visa was tied, which also prevented them from leaving a specific employment contract as they could thus lose their visa. 38 of the participants (20.8%) reported how stressful their work was, and in particular, that they had too much work, and it consequently impacted on their mental health and well-being. Participants felt “overworked (participants 158, 175)”, “stress about the work and how to manage to do all duties (participant 10)”, and reported not getting enough rest, sleep, time to eat, or flexibility to maintain their physical or mental health.

Participants’ feelings of ‘insecurity in relation to their employment’ related to the uncertainty surrounding either finding work or retaining work: “[What is stressful is] my situation today, losing my job and looking for another employer (participant 132).” One participant explained feeling “[stressed] about the future—it’s not clear where I would be in the future (participant 86).”

Overall, within this theme the narratives of respondents highlighted the precariousness of their situations both in relation to their legal status as migrants and their employment, capturing the stresses that resulted from that.

#### Pervasiveness of financial need

Financial needs exacerbate the stresses associated with employment insecurity, and resulted in exploitative situations because of the necessity of prioritising employment. 16 (87.9%) of women described that financial concerns were making them feel stressed, in many cases illustrating that even when women were working long hours and being paid, their wages were not sufficient to sustain them or their families. Women explained, “[My] salary not enough to cover up my and my children’s need (participant 7)” and “I can’t give the amount of money my family needs (participant 63),” a sentiment which was echoed in the responses of many others. Women’s inability to support their families with the salaries they received highlights the interrelationship between this theme and ‘family and obligation’.

#### Family and obligation

The stressors associated with families’ needs may represent a gendered experience, whereby these women experienced obligations both as the carer and breadwinner: “[I feel stressed] when I can’t reach someone at home and I can’t call…When loved ones demand time that I could not give due to working (participant 120).” Women reported stress emanating from worrying about loved ones and being far from family: “When people or my close friends tell how their life or their problem…that makes me feel stressed because I feel that I absorbed their problem (participant 141).” This was evident in other women’s words: “miss my family (participants 34, 106)”; “broken family (participant 37)”; “my children way back home (participant 4).” This theme was very prevalent among the women, with 31 (17.0%) reporting family related stressors.

### Coping strategies

**Fig 2:**
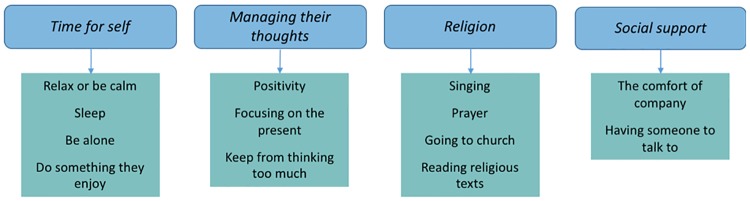
Coping strategies and social support.

Participants were asked what coping strategies they adopt when they are stressed or unhappy, and whether they are effective. Three themes describing coping strategies were identified: *time for self; managing their thoughts*; and *religion* (Fig 2).

#### Time for self

Given the demands of their work life, participants consistently reported a need for *time to themselves* in order to cope with the stressors they encountered daily, both in Singapore, and, more distantly, in their countries of origin. Taking ‘time for self’ included exercise, reading, listening to music, shopping, eating, relaxing, sleeping, and being alone: “I rest a while and make myself calm down (participant 153)”; “I feel ok again to have strength (participant 148)”; “my thoughts can be comforted or kept busy by my activities outside (participant 56).” However, participants reported they were often unable to take time for themselves.

#### Managing their thoughts

Participants’ efforts to cope by *managing their thoughts*, a second theme, included positivity, focusing on the present, and avoiding thinking too much. One participant described she would “keep myself busy and avoid too much thinking (participant 3).” Other participants described they liked to “think of something funny (participant 103),” or “have positive feelings (participant 73).” As one participant explained, “Thinking positively gives me good result (participant 61).” Participants also described how focusing on the present and making the best of things helped them to manage their stress: “As a domestic worker, what to do is to enjoy your housework most of the time…because once in a while, I forget all the things happened around us (participant 72).” Another participant explained, “I just relax and never think so much. I just concentrate on my work (participant 71).”

#### Religion

*Religion*, the third theme, was reported to be an important coping resource. Participants spoke about how singing, prayer, church attendance, and reading religious texts helped them to cope. Across all religions, women described the importance of their faith in addressing stress: “Just pray in a temple (participant 15)”; “Pray and speak to the God where I am believe. To give thanks and to ask for help (participant 34).” When asked if these things helped them to cope, participants’ replies included: “Casting all my anxieties and cares to God help me relax (participant 100)”; “Yes. Because I feel the comfort of God (participant 14)”; “Praying clear my mind and refresh my inner thoughts (participant 165).”

### Social support

Social support was described as a salient coping resource, and two themes were identified: *the comfort of company*, and *having someone to talk to*.

Participants described that *the comfort of company* was beneficial because others gave their understanding, acceptance, comfort, encouragement, love, and physical presence, and that it was helpful for someone to “just be around (participant 96).*” Participants spoke about the importance of having* someone to talk to: “We share each other’s’ concerns. Fellowship (participant 100)”; “By sharing to my friends, give me advice…how to handle such a problem, talking to others is really great (participant 3).” This also included having someone to “listen”, “give advice”, and “make me happy.”

Participants reported the benefits of having someone who would smile or make them smile, tell jokes or a funny story and “make me laugh (participant 98).” The benefits of such social support were obvious in participants’ responses: “Making jokes and fun with me so I can feel less stressed cos I can forget about the problems (participant 52).” Women also described that having someone to encourage their “positive thinking” or provide a positive outlook was helpful. One participant described she liked to “interact with some positive conversation to release any negative thought that weaken the immune system, and to avoid mental disorder (participant 109).”

Eight participants however indicated social support was not helpful: “Just leave me alone and don’t ask what happened. Cos asking why seems rather making me feeling worse and better yet don’t ask what happened with the eyes if it’s obviously sore from crying (participant 120).” Another participant stated, “I don’t need people when I’m stressed (participant 182).”

## Discussion

Psychosocial risks in the workplace are deeply connected with the experience of work-related stress, and have been acknowledged as major public health concerns [17–20]. This study investigated the stressors and coping mechanisms FMDWs in Singapore experienced.

Three hierarchical interrelated themes were identified as causes of stress, including ‘work and agency’, ‘the pervasiveness of financial need’, and ‘family and obligation’. The analysis highlighted how participants’ agency in their workplace is interrelated with ‘the pervasiveness of financial need’. The need for employment to support both themselves and their families contributes to participants’ limited agency, puts them at risk of exploitation, and ultimately presents barriers to being able to take advantage of an entitlement to a rest day [28]. These findings are consistent with previous research in other countries, for example Hong Kong [17] and Spain [18], and point to the multidimensional stressors women experience in relation to both their work environments and lives in host countries, as well as in their countries of origin. The findings highlight the need for further policies to protect FMDWs and ensure that they have agency in their employment and access to support resources [20, 29, 30]. Strategies to evaluate and monitor such policies will also be important to protect FMDWs’ rights.

The participants also described how stressors experienced by their loved ones abroad consequently affected them, a finding that is consistent with previous research on foreign domestic workers and the emotional impact of ‘mothering from a distance’ [23] or labour migration in Sri Lanka, pointing to the impact of distance from family both on migrants and their left-behind families [22]. The interrelationship of *family and obligation* with the other themes also echoes previous research highlighting the politics of reproductive labour. Specifically, it describes the experience among female foreign domestic workers of being caught in the middle of the division of labour between work and home life, and the role that globalisation and the gendered division of labour play in driving these women’s migration [23], and the demand for female foreign domestic workers [35, 36]. The findings of this research, and those in this body of literature are set in the context of and result from the ‘global care chain’, and the gendered experience relating to the demand for reproductive labour that has grown with ‘care transnationalisation’ [2,3].

Coping mechanisms were identified in the research highlighting the need for space and time to cope with stressors, reasonable work hours and pay, and entitlement to a day off. Religion was an important coping resource in the analysis, consistent with a significant body of research on the value and benefit of religion as a way of maintaining a feeling of connectedness to one’s culture [37–39].

The significance of coping mechanisms and women’s resilience has often been overlooked in research on FMDWs due to a focus on their vulnerability. There is limited literature examining the ways in which women, and their strategies to achieve ‘mobility’ or agency, are disempowered in these contexts [40]. Future applied research, interventions and policy work should also be focused around acknowledging, understanding, and supporting the ways in which women cope with these stressors.

Throughout the women’s responses, the social support women described related to forms of ‘emotional support’, for example the role someone played as a confidant or source of advice, rather than ‘instrumental support’, through a physical or tangible source of help or assistance (such as child care, or financial support). Social support was predominantly perceived to be a beneficial resource, consistent with previous research [39–44]. However, it is key to note that there were some women who did not find social support to be a helpful resource, and in some cases preferred not to engage with others when they were feeling stressed, which aligns with recent research suggesting for some groups there may be a paradoxical effect of social support on psychological distress [45]. Among women who highlighted social support as an important coping resource, this may be linked to the loss of social networks and resulting isolation that may occur through migration. FMDWs have been shown to experience high levels of isolation [20], which has been shown to be associated with stress [20], and have a negative effect on psychological health and marginalisation [41–54]. Consequently, it may be beneficial not only to facilitate access to physical resources, but also to foster participants’ access to social support resources. Participants described a range of ways in which they kept connected with their support resources (phone, SMS, and social media), and such information may help in the identification of ways to increase FMDWs’ ability to connect with their communities.

Problematically, the prevention and management of psychosocial risks has not been high on the policy making agenda. While international agencies have helped draw global attention to domestic workers, and resolutions to improve employment conditions for domestic workers worldwide have been adopted (e.g. the International Labour Conference, the Domestic Workers Convention, 2011 (No. 189), and the ILO strategy for action (2011–15)), further efforts are needed to raise awareness of all stakeholders to improve working conditions and employee wellbeing. More progress is needed on integrating healthy workplaces initiatives into policy and practice, devising plans to implement and evaluate the global plan of action on workers’ health, and developing evidence-based policies.

### Strengths and limitations

This study addresses an important gap in research on FMDWs in Singapore, and represents the first empirical data collected on these issues with a large respondent base. Whilst previous studies have often focused on the ‘vulnerability’ of FMDWs based on their increased risk of poor mental health outcomes, our study highlights the coping mechanisms utilised by this population. This is key to developing successful and sustainable initiatives to foster healthy workplaces [16] and to support women in relation to the stressors they experience.

While all participants were proficient in English and did not require the translation resources that were available, we acknowledge that language barriers may still have impacted on their interpretations of the questions and their responses, and consequently the data generated. Further research is needed with populations who experience more significant language barriers, who may be more vulnerable to marginalisation or exploitation due to their literacy and associated factors.

Whilst the study design allowed us to collect free-text qualitative data on the experiences of a large number of women, there is also a need to generate more data for these groups using other qualitative approaches (e.g. in-depth interviews or focus groups) to further deepen understandings of the lived experiences of these groups. Though the responses participants elected to provide were brief, the structure of the free-text questions in the survey allowed us to examine similarities and differences in women’s experiences around the core themes within the framework in our analysis of the qualitative data. However, further in-depth research should also be carried out in this area using other qualitative methods to generate additional data, explore this data using diverse epistemological approaches, and contextualise our findings. The data for the analysis were also limited, as only 100 of the 182 respondents provided free-text data. Whilst there may be differences in experience among those for whom we do not have data, these women had similar socio-demographic backgrounds to those who did provide data, suggesting the data are likely to be representative.

Finally, the results may not be generalisable to FMDWs who did not have access to or were not able to take advantage of rest days. At the time of the research, a similar survey with this group found that 54% of migrant domestic workers reported a weekly day off, however 40% of respondents reported that they had a rest day less than once a week [34]. This population may differ in their financial circumstances, their employment contracts, the demands and restrictions of their workplace, their exposure to exploitation, and the coping resources they can access. Because the study is focused on women, it is also important to consider how gender roles and inequalities may further limit the representation of some populations of FMDWs in research, for example those who are further marginalised by gendered restrictions, violence, or their roles (e.g. caring for children or other family members).

It is also important to acknowledge the influence of the researchers on the findings. The researchers have personal experience of migration, and we recognise that our own experiences of relocating to new countries, leaving behind family members or other loved ones, and learning how to access or develop our own coping mechanisms in relation to stressors may have contributed to our interpretation of the experiences of the immigrant women in the study, and that our experiences are likely to be vastly different. In the analysis, a process of active reflexivity (assessing and documenting the researchers’ perspectives in the research) was undertaken. It is important to recognise that the process of qualitative analysis is interpretative, and requires the researchers’ active and subjective involvement in the production of data. The process of active reflexivity increases the rigour of the research, not by removing the researcher’s influence on the findings or isolate bias (as suggested by a positivist approach assuming a single ‘truth’ exists), but rather to acknowledge multiple realities: the data are grounded in an individual’s experience and informed by the greater context within which they live and the research is conducted.

## Conclusions

This study highlights the stressors FMDWs in Singapore experience, and the coping mechanisms utilised to help address these stressors.

It is important to recognise that this population requires protection in policy not only from deliberate exploitation, but also employment that may be potentially coercive or exploitative due to the socio-economic pressures many FMDWs face and the limited agency that may result from this. Such circumstances could be alleviated by strengthening and expanding the implementation of global frameworks targeted at improving workplace conditions and workers’ rights.

Evaluation and monitoring strategies are also needed to ensure the protection of this population. Such recommendations are in line with key global priorities, for example the Sustainable Development Goals 3, 5, and 8, which emphasise the need to promote wellbeing, gender empowerment, productive employment and decent work.

In relation to the stresses women experience, there is a need to better understand and seek to improve stressors stemming from ‘work and agency’, ‘the pervasiveness of financial need’, and ‘family and obligation’ among FMDWs. The importance of action plans like the WHO ‘Healthy Workplaces’ global model [16], which prioritise efforts to address work-related physical and psychosocial risks, broader social and environmental determinants, and the health of workers, is particularly clear in the findings, in order to protect individual health and achieve key global health targets.

## Supporting information

S1 Table Qualitative dataset. This is the relevant qualitative dataset supporting the findings presented in this manuscript.
(XLSX)

S2 Table COREQ Checklist. This is the completed COREQ Checklist.
(PDF)

## Abbreviations

FMDW: Female Migrant Domestic Worker
MDW: Migrant Domestic Worker
QoL: Quality of Life
